# Predictors of homelessness among vulnerably housed adults in 3 Canadian cities: a prospective cohort study

**DOI:** 10.1186/s12889-016-3711-8

**Published:** 2016-10-03

**Authors:** Matthew J. To, Anita Palepu, Tim Aubry, Rosane Nisenbaum, Evie Gogosis, Anne Gadermann, Rebecca Cherner, Susan Farrell, Vachan Misir, Stephen W. Hwang

**Affiliations:** 1 Centre for Urban Health Solutions, Li Ka Shing Knowledge Institute, St. Michael’s Hospital, 30 Bond Street, Toronto, ON M5B 1W8 Canada; 2 Centre for Health Evaluation and Outcome Sciences, Division of General Internal Medicine, University of British Columbia, Vancouver, BC Canada; 3 University of Ottawa, Ottawa, ON Canada; 4 Royal Ottawa Health Care Group, Ottawa, ON Canada; 5 Division of General Internal Medicine, Department of Medicine, University of Toronto, Toronto, ON Canada

**Keywords:** Homeless persons, Housing, Urban health, Substance-related disorders, Public health

## Abstract

**Background:**

Homelessness is a major concern in many urban communities across North America. Since vulnerably housed individuals are at risk of experiencing homelessness, it is important to identify predictive factors linked to subsequent homelessness in this population. The objectives of this study were to determine the probability of experiencing homelessness among vulnerably housed adults over three years and factors associated with higher risk of homelessness.

**Methods:**

Vulnerably housed adults were recruited in three Canadian cities. Data on demographic characteristics, chronic health conditions, and drug use problems were collected through structured interviews. Housing history was obtained at baseline and annual follow-up interviews. Generalized estimating equations were used to characterize associations between candidate predictors and subsequent experiences of homelessness during each follow-up year.

**Results:**

Among 561 participants, the prevalence of homelessness was 29.2 % over three years. Male gender (AOR = 1.59, 95 % CI: 1.14–2.21) and severe drug use problems (AOR = 1.98, 95 % CI: 1.22–3.20) were independently associated with experiencing homelessness during the follow-up period. Having ≥3 chronic conditions (AOR = 0.55, 95 % CI: 0.33–0.94) and reporting higher housing quality (AOR = 0.99, 95 % CI: 0.97–1.00) were protective against homelessness.

**Conclusions:**

Vulnerably housed individuals are at high risk for experiencing homelessness. The study has public health implications, highlighting the need for enhanced access to addiction treatment and improved housing quality for this population.

## Background

Homelessness is a major public health concern in many communities across North America. Recent reports suggest an estimated 650,000 individuals across the United States and Canada are homeless on any given night [1, 2]. Compared with the general population, homeless individuals have poorer health status and a high prevalence of physical and mental health problems [3–5]. As a result, they experience high rates of healthcare utilization, morbidity, and mortality [5].

Numerous studies have examined risk factors for onset of homelessness and identified several risk factor categories such as demographic characteristics, physical and mental health status, substance use, involvement with the criminal justice system, and housing conditions [6–13]. With regards to demographic factors, younger age has been associated with a higher likelihood of becoming homeless and shorter duration of homelessness [6, 7]. Male gender and African American ethnicity have been identified as independent predictors of homelessness [8, 9]. Obtaining less than a high school education has also been associated with homelessness [6, 10], while being a college graduate has been recognized as a protective factor [11]. Having no income, lower income, or financial difficulties are risk factors for homelessness [10, 12, 13]. Unemployment has been associated with homelessness [11], while employment and earned income are predictors of a shorter duration of homelessness [7].

A variety of physical and mental health conditions have been linked to homelessness [4]. Physical health problems and worsening of general health have been associated with homelessness [6, 11, 14]. Mental illness and family history of mental illness have been identified as predictors of homelessness [12, 13]. Specifically, homelessness has been linked to schizophrenia [8], bipolar disorder [8], anxiety disorder [11], post-traumatic stress disorder [11], and personality disorder [15].

Moreover, substance use and addictions are important risk factors for homelessness [8, 13, 16]. Illicit drug use and having an alcohol use disorder are both predictors of homelessness [6, 15]. Previous research has identified crack cocaine use as a risk factor for becoming and remaining homeless [17]. In addition, recent drug injection use is associated with homelessness [14].

Previous research has also linked housing status and housing conditions such as crowding with homelessness [9]. Living in unstable housing is also a predictor of homelessness [12]. Protective factors against homelessness include receipt of subsidized housing and having one’s own place [9]. Preliminary research has also found an association between social support and homelessness, suggesting that lower social support is linked to chronic homelessness [18].

Although previous research has identified risk factors for onset of homelessness, few studies have examined risk factors for homelessness among individuals who are vulnerably housed, which has been defined as experiencing prior homelessness or having frequent housing transitions [19]. Emerging research suggests that vulnerably housed individuals have similar health and social outcomes to homeless individuals and are at an increased risk of experiencing homelessness [9, 19].

Recent studies have found that a substantial proportion of homeless individuals who obtain housing subsequently experience a recurrence of homelessness [20, 21]. In one study of 344 single adults in emergency shelters in New York City who were newly homeless, 24 % of those who obtained housing (81 %) over an 18-month period had another episode of homelessness. Recurrent homelessness was more common among those who were initially rehoused with family and those with a high school education [20]. Compared to housed individuals, those experiencing recurrent homelessness were more likely to have a 30-day and lifetime history of alcohol and substance use disorders [20]. Another U.S. study examined predictors of returning to homelessness after attaining housing in a sample of 392 formerly homeless veterans who participated in a trial of case management and rent subsidies, case management only, or standard care [21]. Over the course of a five year period, 44 % of all participants experienced another homeless episode after being housed. Participants who received case management and rent subsidies had significantly longer periods of continuous housing compared with participants in the two other groups [21].

Given that vulnerably housed individuals are at risk of experiencing episodes of homelessness, it is important to identify potentially predictive factors linked to subsequent homelessness in this population. The present paper reports findings from the Health and Housing in Transition (HHiT) study, a prospective cohort study that tracked the health and housing status of homeless and vulnerably housed adults in three major Canadian cities [19]. The objectives of this paper are to examine participants who were vulnerably housed at the baseline interview and determine the probability of experiencing homelessness over a three-year follow-up period and the individual characteristics associated with higher risk of homelessness.

## Methods

### Participants

Homeless and vulnerably housed persons aged 18 or older who were single (i.e. not living with a partner or dependent child) were recruited in Ottawa, Toronto, and Vancouver from January to December 2009. Homelessness was defined as living within the last seven days at a shelter, public space, vehicle, abandoned building, or someone else’s home, and not having a home of one’s own. Vulnerably housed was defined as currently living in one’s own room or apartment, but having been homeless or had two or more moves in the past twelve months. Full-time students and individuals who were visiting the city for three months or less were excluded.

### Recruitment

The sampling procedure for recruiting homeless participants was based on the design suggested by Ardilly and Le Blanc (2001) [22]. Study participants were recruited at homeless shelters and meal programs. Homeless participants who did not use shelters were recruited at meal programs proportionally to the estimated number of homeless persons that slept on the street in each respective city. Vulnerably housed participants were recruited from randomly selected rooming houses in Ottawa and Toronto, and from Single Room Occupancy (SRO) hotels in Vancouver. Due to difficulties in gaining access to some of these locations, the recruitment strategy for vulnerably housed individuals was modified to include meal programs, drop-in centers, and community health centers. Data were collected from participants between January 2009 and February 2013. All study participants provided written informed consent and received $20 CDN upon interview completion. The Research Ethics Boards at the University of Ottawa; St. Michael’s Hospital, Toronto; and the University of British Columbia, Vancouver approved this study.

### Survey instrument

Full details of all survey instruments used in the study have been reported elsewhere [19]. Data on demographic characteristics, health conditions and health status, alcohol and drug use, housing history and quality, social support, legal incidents, and victimization were collected using structured, in-person interviews conducted by trained research personnel immediately following recruitment. Interviews took approximately 60 to 90 minutes to complete. Participants reported their ethnic background based on categories adapted from the Statistics Canada Ethnic Diversity Survey [23].

Chronic health conditions listed in the survey tool were adapted from the Canadian Community Health Survey [24], and participants were asked to report any chronic health conditions that had lasted or were expected to last six months or more and had been diagnosed by a healthcare professional. Lifetime prevalence of mental health diagnoses was determined by self-report. Lifetime prevalence of traumatic brain injury (TBI) was determined using a question from a previous study on prison inmates [25]. Participants were asked whether they had ever had “an injury to the head which knocked you out or at least left you dazed, confused, or disoriented?” Health status was determined using the 12-item Short Form Health Survey (SF-12) to generate Physical Component Summary (PCS) and Mental Component Summary (MCS) subscale scores [26].

Alcohol use was assessed using the Alcohol Use Disorders Identification Test (AUDIT), with a score of eight or more resulting in a positive screen, with scores of 8–15 indicating hazardous, 16–19 harmful, and 20–40 indicating high levels of risk related to alcohol [27]. Drug use problems experienced by participants were assessed using the 10-item version of the Drug Abuse Screening Test (DAST-10) [28]. Scores of three or higher on the DAST-10 resulted in a positive screen, with higher scores indicating moderate (scores 3–5), substantial (scores 6–8), or severe (scores 9–10) drug use problems. The Housing Quality Score was used to determine self-reported quality of the current living environment in 6 domains: comfort, safety, spaciousness, privacy, friendliness, and overall quality [29]. Each item was ranked on a 7-point Likert scale with a maximum total score of 42. Social support was assessed using the Social Support Network Inventory (SSNI), a questionnaire that measured the size of a person’s social network and perceived social support [30].

Housing history data were categorized based on methods adapted from Tsemberis et al. [31]. Each residence in a participant’s housing history was classified into one of 25 types of residence, which were then classified into one of three mutually exclusive residence categories: housed, institution, and homeless. Periods of time spent in institutions were considered periods of being homeless or housed based on a functional classification [31]. Further details are available from the authors upon request.

Participants provided contact information during administration of the baseline survey so that they could be located for follow-up surveys administered approximately one year, two years, and three years after the baseline survey. The follow-up survey included questions of a similar nature to the baseline survey on health status and health conditions, alcohol and drug use, housing status and quality, and social support.

### Data analysis

Vulnerably housed participants originally recruited into the study who did not complete any follow-up interviews were excluded from the analyses. Descriptive statistics were used to summarize all quantitative variables. The percentage of vulnerably housed adults who experienced homelessness anytime over the three-year follow-up period was calculated. The main outcome of interest was whether a vulnerably housed participant ever experienced homelessness during any of the three one-year periods between the baseline and follow-up 1 interviews, the follow-up 1 and follow-up 2 interviews, and the follow-up 2 and follow-up 3 interviews.

Baseline characteristics were summarized using means, standard deviations, medians, interquartile ranges, and proportions, wherever appropriate. Comparisons between vulnerably housed participants who did and did not experience homelessness during the three-year follow-up period were performed for baseline characteristics. *P*-values were calculated from *t*-test or Wilcoxon rank-sum test for continuous variables. Chi-square test or Fisher’s exact test were used for categorical variables.

Various demographic, health, and housing variables were assessed for an association with a higher probability of becoming homeless over a three-year follow-up period. These characteristics included fixed covariates (determined at the baseline interview) and time-varying covariates (determined at baseline, follow-up 1, follow-up 2 interviews).

The list of candidate predictors of homelessness was developed based on a literature review and consultation with experts. Characteristics assessed for association with experiencing homelessness in the follow-up period were city, time interval, and 1) fixed predictors including: age, gender, ethnicity, highest level of education, percentage of time spent homeless two years prior to baseline divided by 10 for ease of interpretation, number of chronic health conditions (≥3 versus <3), history of a mental health diagnosis, history of TBI, and 2) time-varying predictors evaluated at the beginning of each one-year interval including: employment in the past 12 months, total income in the past 12 months, SF-12 PCS, SF-12 MCS, AUDIT risk level, DAST risk level, housing quality score, and social support network size. For example, SF-12 PCS at baseline, follow-up 1, and follow-up 2 interviews was a time-varying predictor for the main outcome of homelessness during the periods between the baseline and follow-up 1 interviews, the follow-up 1 and follow-up 2 interviews, and the follow-up 2 and follow-up 3 interviews.

Generalized estimating equations (GEE) with the logit link were used to determine the association between predictors and experiencing homelessness, accounting for the correlations between repeated measurements (SAS PROC GENMOD). For fixed predictors, the quasi-likelihood information criteria (QIC) were used to find the correlation structure. For time-varying predictors, we applied the Rotnitzky and Jewell approach [32, 33], and chose the correlation structure for which its associated empirical covariance matrix was closer to the model-based covariance matrix. The GEE model was developed in two steps. Step 1 included city, time interval and fixed predictors, which were retained in the model if significantly associated with or clinically relevant for the outcome (Core Model); Step 2 added time-varying predictors to the Core Model, one at a time. Time-varying predictors significantly associated with or clinically relevant for the outcome were retained in the final model. Analyses for the 2-step process were performed using the exchangeable working correlation structure and coding of time as a continuous variable (time interval years 1, 2, 3) because these settings yielded slightly better goodness of fit statistics. All statistical tests were two-tailed and statistical significance was set at a *P*-value of 0.05 or less. SAS 9.4 (SAS Institute, Inc., Cary, NC) was used for all analyses.

## Results

Of 594 vulnerably housed individuals interviewed at baseline, 561 (94.4 %) completed at least one of three follow-up interviews and were included in the analyses. Attrition was due to inability to locate participants, refusal to participate, and death. Baseline characteristics for the whole sample and stratified by city are provided in Table 1.

**Table 1:** Baseline characteristics of 561 vulnerably housed adults in 3 Canadian cities

Characteristic	Total (*N* = 561)^a^ n (%)	Ottawa (*N* = 190)^a^ n (%)	Toronto (*N* = 186)^a^ n (%)	Vancouver (*N* = 185)^a^ n (%)
Age Group				
< 30 years	63 (11.2)	30 (15.8)	12 (6.5)	21 (11.4)
30–39 years	137 (24.4)	50 (26.3)	40 (21.5)	47 (25.4)
40–49 years	221 (39.4)	62 (32.6)	87 (46.8)	72 (38.9)
≥ 50 years	140 (25)	48 (25.3)	47 (25.3)	45 (24.3)
Mean age (SD)	42.6 (9.8)	41.5 (10.5)	43.8 (9.4)	42.5 (9.4)
Gender				
Male	391 (69.7)	149 (78.4)	125 (67.2)	117 (63.2)
Female	162 (28.9)	41 (21.6)	57 (30.7)	64 (34.6)
Transgendered	8 (1.4)	0	4 (2.2)	4 (2.2)
Ethnicity				
White	344 (63.4)	149 (79.3)	90 (51.1)	105 (58.7)
Black/African-Canadian	36 (6.6)	3 (1.6)	27 (15.3)	6 (3.4)
First Nations/Aboriginal	122 (22.5)	32 (17)	37 (21)	53 (29.6)
Mixed/other	41 (7.6)	4 (2.1)	22 (12.5)	15 (8.4)
Born in Canada	496 (89.4)	183 (96.8)	147 (79)	166 (92.2)
Highest level of education				
Some high school	279 (50.4)	100 (53.2)	86 (46.7)	93 (51.1)
Completed high school/equivalent	121 (21.8)	36 (19.2)	42 (22.8)	43 (23.6)
Some post-secondary or higher	154 (27.8)	52 (27.7)	56 (30.4)	46 (25.3)
Partnered	134 (24.3)	48 (25.7)	37 (20.1)	49 (27.1)
Employed in past 12 months	213 (38)	84 (44.4)	61 (32.8)	68 (36.8)
Monthly income, median (IQR)	900 (600–1320)	912.33 (591–1340)	750 (550–1200)	966 (698–1480)
Percent of income spent on rent, median (IQR)	41.4 (25.0–62.0)	43.0 (27.3–63.6)	38.9 (22.8–65.0)	41.1 (27.8–57.9)
Chronic health conditions				
0	55 (9.8)	10 (5.3)	36 (19.4)	9 (4.9)
1	106 (18.9)	31 (16.3)	38 (20.4)	37 (20)
2	97 (17.3)	32 (16.8)	36 (19.4)	29 (15.7)
≥ 3	303 (54)	117 (61.6)	76 (40.9)	110 (59.5)
History of a mental health diagnosis	303 (54.9)	120 (64.9)	78 (42.2)	105 (57.7)
History of traumatic brain injury	358 (64)	136 (72)	97 (52.4)	125 (67.6)
SF-12 PCS, mean (SD)	43.6 (10.8)	43.01 (11.3)	44.42 (10.26)	43.35 (10.8)
SF-12 MCS, mean (SD)	39.93 (13)	39.04 (13.5)	40.77 (12.79)	39.97 (12.76)
Pregnancy in past 12 months	12 (7.5)	4 (10)	3 (5.3)	5 (7.8)
Currently smoking				
Daily	451 (80.8)	161 (85.2)	138 (74.6)	152 (82.6)
Occasionally	46 (8.2)	11 (5.8)	22 (11.9)	13 (7.1)
Not at all	61 (10.9)	17 (9)	25 (13.5)	19 (10.3)
Positive AUDIT screen	222 (39.7)	82 (43.4)	76 (40.9)	64 (34.8)
AUDIT risk				
Low	337 (60.3)	107 (56.6)	110 (59.1)	120 (65.2)
Hazardous	88 (15.7)	31 (16.4)	30 (16.1)	27 (14.7)
Harmful	31 (5.6)	12 (6.4)	11 (5.9)	8 (4.4)
High	103 (18.4)	39 (20.6)	35 (18.8)	29 (15.8)
Positive DAST screen	322 (57.6)	109 (57.7)	85 (45.7)	128 (69.6)
DAST risk				
No drug use in past 12 months	136 (24.3)	35 (18.5)	72 (38.7)	29 (15.8)
Low	101 (18.1)	45 (23.8)	29 (15.6)	27 (14.7)
Moderate	141 (25.2)	50 (26.5)	34 (18.3)	57 (31)
Substantial	134 (24)	37 (19.6)	38 (20.4)	59 (32.1)
Severe	47 (8.4)	22 (11.6)	13 (7)	12 (6.5)
Arrests and/or incarceration in past 12 months	190 (34)	68 (36.2)	61 (32.8)	61 (33)
Physical assault victim in past 12 months	206 (37)	74 (39.6)	63 (34.1)	69 (37.3)
Sexual assault victim in past 12 months	46 (8.3)	14 (7.5)	11 (5.9)	21 (11.5)
Age at first homelessness, median (IQR)	24 (16–38)	22 (16–38)	27 (16–39)	23 (15–38)
Lifetime years of homelessness, median (IQR)	3.15 (1–6.73)	2.38 (0.93–6)	3.5 (1–8.56)	3.56 (1.38–6.74)
Own place	511 (95.7)	182 (97.3)	155 (91.2)	174 (98.3)
Subsidized housing	213 (40.8)	42 (23.2)	97 (57.1)	74 (43.3)
Residence Type				
Own house, apartment	180 (32.1)	66 (34.7)	99 (53.2)	15 (8.1)
Stay with friends and/or relatives	13 (2.3)	2 (1.1)	9 (4.8)	2 (1.1)
Rooming house	197 (35.1)	118 (62.1)	75 (40.3)	4 (2.2)
SRO	160 (28.5)	0	0	160 (86.5)
Substance abuse treatment facility	4 (0.7)	0	0	4 (2.2)
Halfway house	2 (0.4)	2 (1.1)	0	0
Supportive housing	1 (0.2)	1 (0.5)	0	0
Alternative housing	3 (0.5)	0	3 (1.6)	0
Other	1 (0.2)	1 (0.5)	0	0
Housing quality, mean (SD)	27.53 (8.2)	27.44 (8.6)	28.48 (8.1)	26.67 (7.85)
Social support network size				
0	95 (17.3)	33 (17.8)	36 (19.4)	26 (14.5)
1	61 (11.1)	21 (11.4)	16 (8.6)	24 (13.4)
2–3	104 (18.9)	29 (15.7)	38 (20.4)	37 (20.7)
≥ 4	290 (52.7)	102 (55.1)	96 (51.6)	92 (51.4)

^a^Percentages based on complete data; percentages provided by column

As per design, equal numbers of vulnerably housed participants were recruited in each city (34 %, 33 %, 33 % in Ottawa, Toronto, and Vancouver, respectively). The mean age was 42.6 (standard deviation [SD] 9.8) years, and the majority of participants were male, White, born in Canada, had completed some high school, not partnered, and currently unemployed. Participants had a median monthly income of $900 CDN (interquartile range [IQR] 600–1320) with a median percentage of income spent on rent equal to 41.4 % (IQR 25–62 %).

More than half of vulnerably housed participants (54 %) were living with 3 or more chronic health conditions. Fifty-five percent of participants had ever received a mental health diagnosis and 64 % reported having a prior TBI. Mean SF-12 PCS score was 43.6 (SD 10.79) and mean SF-12 MCS score was 39.93 (SD 13.02). Two hundred and twenty-two (40 %) participants had a positive AUDIT screen and 322 (58 %) had a positive DAST screen. Almost all participants had their own place (95.7 %) or stayed in a place belonging to friends or family (4.3 %). Of this group, 85 % had experienced homelessness in the one-year period before entering the study. Median age at first homelessness was 24 (IQR 16–38) and median lifetime years of homelessness was 3.15 (IQR 1–6.73) among participants. Less than half of participants (41 %) were residing in subsidized housing. Mean housing quality score was 27.53 (SD 8.21).

Over the three-year period, 269 of 561 (48 %) participants experienced at least one episode of homelessness, while 292 (52 %) never reported homelessness. Among those who had experienced homelessness, the median duration was 202 (IQR 92–456) days. Those who experienced homelessness during the three-year follow-up period were significantly more likely at baseline to have been younger, born in Canada, completed high school/equivalent, employed in the past 12 months, smoked cigarettes daily, have positive AUDIT and DAST screens, and experienced arrests or incarceration in the past 12 months (p < 0.05) (Table 2).

**Table 2:** Baseline characteristics for 561 vulnerably housed participants across 3 Canadian cities who did and did not experience homelessness during the 3-year follow-up period

Characteristic	Total (*N* = 561)^a^ n (%)	Ever homeless (*N* = 269)^a^ n (%)	Never homeless (*N* = 292)^a^ n (%)	*P*-value^b^
Age Group				0.0226
< 30 years	63 (11.2)	31 (11.5)	32 (11.0)	
30–39 years	137 (24.4)	77 (28.6)	60 (20.6)	
40–49 years	221 (39.4)	108 (40.2)	113 (38.7)	
≥50 years	140 (25)	53 (19.7)	87 (29.8)	
Mean age (SD)	42.6 (9.8)	41.3 (9.2)	43.8 (10.2)	0.0033
Gender				0.0527
Male	391 (69.7)	65 (24.2)	97 (33.2)	
Female	162 (28.9)	200 (74.4)	191 (65.4)	
Transgendered	8 (1.4)	4 (1.5)	4 (1.4)	
Ethnicity				0.7361
White	344 (63.4)	167 (64.5)	177 (62.3)	
Black/African-Canadian	36 (6.6)	14 (5.4)	22 (7.8)	
First Nations/Aboriginal	122 (22.5)	59 (22.8)	63 (22.2)	
Mixed/other	41 (7.6)	19 (7.3)	22 (7.8)	
Born in Canada	496 (89.4)	246 (92.5)	250 (86.5)	0.0225
Highest level of education				0.0009
Some high school	279 (50.4)	119 (44.7)	160 (55.6)	
Completed high school/equivalent	121 (21.8)	76 (28.6)	45 (15.6)	
Some post-secondary or higher	154 (27.8)	71 (26.7)	83 (28.8)	
Partnered	134 (24.3)	64 (24.2)	70 (24.4)	0.9478
Employed in past 12 months	213 (38.0)	114 (42.4)	99 (34.0)	0.0418
Monthly income, median (IQR)	900 (600–1320)	920.0 (630–1540)	889 (586–1200)	0.0906
Percent of income spent on rent, median (IQR)	41.4 (25.0–62.0)	41.0 (22.5–62.3)	41.7 (27.3–60.9)	0.4403
Chronic health conditions				0.0609
0	55 (9.8)	32 (11.9)	23 (7.9)	
1	106 (18.9)	52 (19.3)	54 (18.5)	
2	97 (17.3)	54 (20.1)	43 (14.7)	
≥3	303 (54)	131 (48.7)	172 (58.9)	
History of a mental health diagnosis	303 (54.9)	153 (57.3)	150 (52.6)	0.2703
History of traumatic brain injury	358 (64)	169 (62.8)	189 (65.2)	0.5634
SF-12 PCS, mean (SD)	43.6 (10.8)	44.3 (11.2)	43.0 (10.4)	0.1507
SF-12 MCS, mean (SD)	39.9 (13.0)	40.3 (12.4)	39.6 (13.6)	0.5231
Pregnancy in past 12 months	12 (7.5)	6 (9.2)	6 (6.3)	0.4798
Currently smoking				0.0002
Daily	451 (80.8)	231 (86.2)	220 (75.9)	
Occasionally	46 (8.2)	23 (8.6)	23 (7.9)	
Not at all	61 (10.9)	14 (5.2)	47 (16.2)	
Positive AUDIT screen	222 (39.7)	119 (44.2)	103 (35.5)	0.0353
AUDIT risk				0.1388
Low	337 (60.3)	150 (55.8)	187 (64.5)	
Hazardous	88 (15.7)	44 (16.4)	44 (15.2)	
Harmful	31 (5.6)	16 (6.0)	15 (5.2)	
High	103 (18.4)	59 (21.9)	44 (15.2)	
Positive DAST screen	322 (57.6)	169 (62.8)	153 (52.8)	0.0161
DAST risk				0.0994
No drug use in past 12 months	136 (24.3)	57 (21.2)	79 (27.2)	
Low	101 (18.1)	43 (16.0)	58 (20.0)	
Moderate	141 (25.2)	68 (25.3)	73 (25.2)	
Substantial	134 (24)	76 (28.3)	58 (20.0)	
Severe	47 (8.4)	25 (9.3)	22 (7.6)	
Arrests and/or incarceration in past 12 months	190 (34)	108 (40.3)	82 (28.2)	0.0025
Physical assault victim in past 12 months	206 (37)	99 (37.1)	107 (36.9)	0.9645
Sexual assault victim in past 12 months	46 (8.3)	26 (9.7)	20 (6.9)	0.2335
Age at first homelessness, median (IQR)	24 (16–38)	22.0 (16–36)	25 (16–40)	0.1910
Lifetime years of homelessness, median (IQR)	3.2 (1.0–6.7)	3.0 (1.0–6.7)	3.3 (1.0–6.7)	0.6692
Own place	511 (95.7)	240 (94.5)	271 (96.8)	0.1915
Subsidized housing	213 (40.8)	91 (36.6)	122 (44.7)	0.0587
Residence Type				0.2207
Own house, apartment	180 (32.1)	89 (33.1)	91 (31.2)	
Stay with friends and/or relatives	13 (2.3)	8 (3.0)	5 (1.7)	
Rooming house/SRO	357 (63.6)	164 (61.0)	193 (66.1)	
Institution/Other	11 (2.0)	8 (3.0)	3 (1.0)	
Housing quality, mean (SD)	27.53 (8.2)	27.4 (8.2)	27.7 (8.2)	0.6849
Social support network size				0.3289
0	95 (17.3)	45 (17.0)	50 (17.5)	
1	61 (11.1)	23 (8.7)	38 (13.3)	
2–3	104 (18.9)	50 (18.9)	54 (19.0)	
≥4	290 (52.7)	147 (55.5)	143 (50.2)	

^a^Percentages based on complete data; percentages provided by column

^b^ *P*-value calculated from *t*-test or Wilcoxon rank-sum test for continuous variables, chi-square test or Fisher’s exact test for categorical variables

**Fig. 1:**
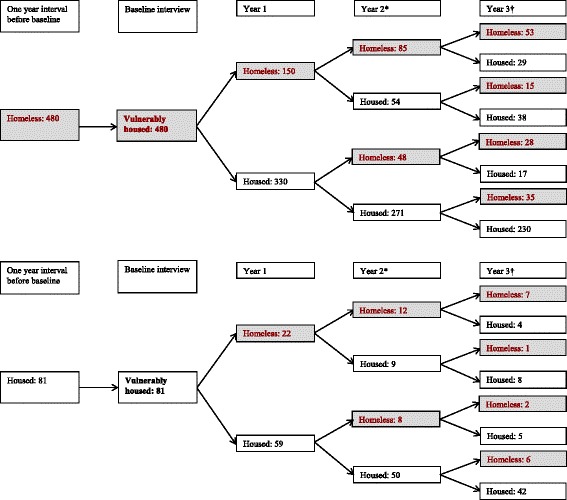
Housing transitions for 561 vulnerably housed participants in 3 Canadian cities over a 3-year follow-up period

Across 1618 residential records of the 561 participants, the prevalence of homelessness was 29 %. Specifically, the prevalence of homelessness among residential records of participants was 30.7 %, 28.5 % and 28.3 % between baseline and follow-up at 1 year, between follow-up at 1 year and follow-up at 2 years, between follow-up at 2 years and follow-up at 3 years, respectively. Participants experienced a variety of residential state trajectories, where many individuals experienced transitions into and out of homelessness (Fig. 1).

Table 3 presents GEE results. In the final multivariable model, residing in Ottawa (Adjusted Odds Ratio [AOR] = 1.88, 95 % Confidence Interval [CI]: 1.31–2.70), male gender (AOR = 1.59, 95 % CI: 1.14–2.21), percentage of time homeless prior to the baseline interview (AOR = 1.06, 95 % CI: 1.01–1.11), moderate DAST risk level (AOR = 1.4, 95 % CI: 1.00–1.97) substantial DAST risk level (AOR = 1.71, 95 % CI: 1.20–2.44), and severe DAST risk level (AOR = 1.98, 95 % CI: 1.22–3.20) were independently associated with experiencing homelessness over the three-year follow-up period. Factors that were independently associated with a decreased likelihood of experiencing homelessness over the three-year follow-up period were having less than a high school education (AOR = 0.68, 95 % CI: 0.50–0.91), having 3 or more chronic health conditions (AOR = 0.55, 95 % CI: 0.33–0.94), and higher per unit housing quality (AOR = 0.99, 95 % CI: 0.97–1.00).

**Table 3:** Multivariable GEE logistic regression model of characteristics associated with experiencing homelessness during a 3-year follow-up period among vulnerably housed participants in 3 Canadian cities

Characteristic	Core Model Adjusted Odds Ratio (95 % CI)	Final Model Adjusted Odds Ratio (95 % CI)	*P* value
City			
Ottawa	1.66 (1.16–2.37)	1.88 (1.31–2.70)	<0.001
Toronto	1.04 (0.71–1.54)	1.15 (0.77–1.71)	0.497
Vancouver	1	1	--
For every year of age	0.98 (0.97–1.00)	0.99 (0.97–1.00)	0.126
Gender			
Male	1.64 (1.19–2.28)	1.59 (1.14–2.21)	0.007
Female	1	1	--
Highest level of education			
Less than high school	0.65 (0.48–0.87)	0.68 (0.50–0.91)	0.009
More than high school	1	1	--
Chronic conditions			
1	0.66 (0.38–1.15)	0.63 (0.35–1.11)	0.110
2	0.81 (0.47–1.41)	0.83 (0.47–1.45)	0.505
≥3	0.60 (0.36–1.01)	0.55 (0.33–0.94)	0.029
None	1	1	--
Per 10 % of time spent homeless prior to baseline	1.07 (1.02–1.12)	1.06 (1.01–1.11)	0.018
Per interval year of follow-up	0.96 (0.85–1.08)	0.96 (0.85–1.09)	0.515
Per unit housing quality	--	0.99 (0.97–1.00)	0.048
DAST risk level			
Low	--	1.29 (0.90–1.84)	0.164
Moderate	--	1.40 (1.00–1.97)	0.049
Substantial	--	1.71 (1.20–2.44)	0.006
Severe	--	1.98 (1.22–3.20)	0.003
No drug use	--	1	--

## Discussion

Among residential records of vulnerably housed participants, the prevalence of homelessness was 29 % over the three-year follow-up period, suggesting that experiences of homelessness are relatively common in this population. This finding is similar to other studies that have found that 24–44 % of individuals with prior homeless episodes experienced a recurrence of homelessness [20, 21]. The current study also revealed a variety of housing trajectories among participants, with many individuals experiencing multiple episodes of homelessness and being housed over the follow-up period. These findings suggest that vulnerably housed individuals frequently experience housing instability and housing transitions, similar to what has been previously reported for homeless individuals [19].

The study also identified several risk factors of subsequent homelessness. Vulnerably housed individuals who were male, residing in Ottawa, had spent a higher percentage of time homeless prior to the baseline interview, or had moderate to severe drug use problems were significantly more likely to experience homelessness over the three-year follow-up period despite adjustment for potential confounders.

Gender was a significant predictor of homelessness among vulnerably housed individuals, with men being 1.6 times more likely to experience homelessness compared to women during the follow-up period. This is consistent with previous research that has identified male gender as a risk factor for homelessness [8]. Ottawa participants were also more likely to experience homelessness during the follow-up period compared to individuals living in Vancouver. The reasons for this observation are unclear, but these findings may be attributed to differences that could not be captured between participants at different study sites and the availability of housing and social services in each respective city. Similar to prior studies [20, 21], those who reported a higher proportion of time spent homeless before the baseline interview were also more likely to experience homelessness during the follow-up period.

Moderate to severe drug use problems were independently associated with experiencing homelessness in the follow-up period, with severe drug use problems significantly associated with the greatest likelihood of homelessness. Those who reported severe drug use problems were almost two times more likely to experience homelessness during the follow-up period compared with participants who reported no drug use. These findings are consistent with previous research that identified drug use as the greatest risk factor for housing instability [21]. Findings from the current study additionally suggest a dose-dependent relationship of drug use and homelessness, with more severe drug use problems being linked to an increased likelihood of subsequent homelessness among vulnerably housed adults.

Despite adjustment, participants who had less than a high school education, had 3 or more chronic health conditions, or reported higher housing quality were significantly less likely to experience homelessness over the follow-up period. While the association between attaining less than a high school education and decreased likelihood of homelessness was unexpected [6], it is similar to a previous study which found that high school completion was associated with recurrent homelessness among adults who had experienced prior homelessness [20]. The underlying explanation for this finding is unclear, but may in part, be due to the higher likelihood of individuals with lower education levels to have a learning disorder or experience unemployment and subsequently qualify for social assistance programs. Paradoxically, these vulnerably housed individuals may be less likely to experience homelessness while those with a high school education may be eligible for more employment opportunities, but may also be more likely to experience recurrent homelessness [20].

Vulnerably housed adults who had 3 or more chronic health conditions were less likely to experience homelessness over the follow-up period. This finding could also be attributed to the fact that individuals living with multiple chronic conditions may be more likely to be eligible for financial assistance programs and access to health and social services. They may also be given priority for subsidized and supportive housing because of their chronic medical conditions.

Those who reported higher housing quality were also less likely to experience homelessness over the follow-up period. This is an important finding that has not been previously reported and has implications for efforts to prevent homelessness. It appears that individuals living in higher quality housing characterized by aspects such as comfort, safety, privacy, and spaciousness were more likely to remain in that housing. Previous research suggests that housing quality concerns are common among vulnerably housed populations, with up to 85 % of affordable housing properties having at least one health-related housing quality issue [34]. Our findings suggest that low housing quality may be a modifiable protective factor for homelessness. Thus, improving the quality of low-cost housing may decrease the likelihood that vulnerably housed individuals become homeless in the future.

The study has service provision and public health implications, highlighting the prevalence of homelessness among vulnerably housed individuals and the need for screening and treating modifiable risk factors among this population. Specifically, the findings highlight the importance of connecting individuals with addictions treatment to potentially reduce the risk of subsequent homelessness. The study also found that housing quality may be a protective factor against homelessness among vulnerably housed individuals, suggesting that this population may benefit from targeted efforts to improve housing quality in domains such as comfort, privacy, spaciousness, and safety which in turn, may help prevent subsequent homelessness. This can include assisting individuals to access subsidized housing and rent supplements to achieve housing stability [9, 35].

### Limitations

The study has several limitations which may restrict the interpretation of its findings. The study sample was limited to single adults and the sampling strategy may not have been fully representative of the entire vulnerably housed population. Since data on demographic characteristics, health conditions, and housing were collected by self-report, accuracy may have been affected by recall and other sources of reporting bias. The study did not examine personality traits of participants which could contribute to both increased risk of substance use and likelihood of experiencing homelessness. In addition, participants who had severe drug use problems may not have completed the survey. Thus, the relationship between drug use problems and subsequent homelessness may have revealed an even stronger association.

Future studies should investigate risk factors for recurrent homelessness and examine predictors of homelessness over a longer follow-up period. In addition, interventions to prevent homelessness among vulnerably housed individuals should be explored given the potential public health implications.

## Conclusions

The study followed housing trajectories of vulnerably housed adults and found that 29 % of the study sample experienced homelessness over a 3-year period. The study also identified risk factors for homelessness among vulnerably housed individuals such as male gender, higher percentage of time spent homeless prior to baseline, and moderate to severe drug use problems. Protective factors included having 3 or more chronic conditions and higher housing quality. The study has important public health implications, highlighting the need for addictions treatment and efforts to improve housing quality among vulnerably housed individuals, which may help prevent subsequent experiences of homelessness in this population.

## Abbreviations

AOR: Adjusted odds ratio
AUDIT: Alcohol Use Disorders Identification Test
DAST-10: 10-item Drug Abuse Screening Test
GEE: Generalized estimating equations
IQR: Interquartile range
MCS: Mental Component Summary
PCS: Physical Component Summary
SD: Standard deviation
SF-12: 12-item Short Form Health Survey
SRO: Single room occupancy
SSNI: Social Support Network Inventory
TBI: Traumatic brain injury
